# Cryo-electron microscopy in the fight against COVID-19—mechanism of virus entry

**DOI:** 10.3389/fmolb.2023.1252529

**Published:** 2023-10-06

**Authors:** Satish Bodakuntla, Christopher Cyrus Kuhn, Christian Biertümpfel, Naoko Mizuno

**Affiliations:** ^1^ Laboratory of Structural Cell Biology, National Heart Lung and Blood Institute, National Institutes of Health, Bethesda, MD, United States; ^2^ National Institute of Arthritis and Musculoskeletal and Skin Diseases, National Institutes of Health, Bethesda, MD, United States

**Keywords:** cryo-EM, cryo-ET, SARS-CoV-2, COVID-19, spike protein, ACE2

## Abstract

Cryogenic electron microscopy (cryo-EM) and electron tomography (cryo-ET) have become a critical tool for studying viral particles. Cryo-EM has enhanced our understanding of viral assembly and replication processes at a molecular resolution. Meanwhile, *in situ* cryo-ET has been used to investigate how viruses attach to and invade host cells. These advances have significantly contributed to our knowledge of viral biology. Particularly, prompt elucidations of structures of the SARS-CoV-2 spike protein and its variants have directly impacted the development of vaccines and therapeutic measures. This review discusses the progress made by cryo-EM based technologies in comprehending the severe acute respiratory syndrome coronavirus-2 (SARS-Cov-2), the virus responsible for the devastating global COVID-19 pandemic in 2020 with focus on the SARS-CoV-2 spike protein and the mechanisms of the virus entry and replication.

## Cryo-electron microscopy and tomography for viral studies

Over the past decades, cryo-electron microscopy (cryo-EM) has emerged as a valuable method for obtaining structural information about biological samples and elucidating intricate biochemical and cellular processes. Central to this success is the continuing advances in utilizing novel hardware components and computational approaches (Nogales and Scheres, 2015; Egelman, 2016; Maruthi et al., 2020). Particularly, the utilization of direct detection device (DDD) cameras with increased readout frequency, allowed to acquire dose-fractionated multi-frame movies, which enabled to correct beam-induced sample motions and enhance the extraction of high-resolution information (Zheng et al., 2017). On the software side, new data processing algorithms including deep learning approaches for particle picking and implementation of automated pipelines (Scheres, 2012; Punjani et al., 2017; Bepler et al., 2019) have further bolstered the structural biology field. As a result of these developments, there has been an exponential increase in the publication of high-resolution maps in recent years, marking a new era in the cryo-EM field, stated as the “Resolution Revolution” (Kuhlbrandt, 2014). Cryo-EM technology now faces a new challenge: visualizing molecular architecture in their cellular environments using *in situ* cryo-electron tomography (cryo-ET). In cryo-ET, instead of extracting biomolecular complexes from their hosts or reconstituting the complexes from recombinantly prepared proteins, the molecular assemblies of intracellular components are imaged directly in their native environments. During the data collection, the area of interest is incrementally tilted, resulting in a stack of projections from various angular perspectives. This stack of images is computationally aligned and reconstructed into a 3D volume, revealing the ultrastructural composition behind the biological processes (Beck and Baumeister, 2016; Turk and Baumeister, 2020).

Emerging technologies like cryo-EM and cryo-ET have become an essential pillar to elucidate the macromolecular complexes such as viral particles and even viruses themselves (De Rosier and Klug, 1968; Forster et al., 2005; Mangala Prasad et al., 2017; Ke et al., 2020; Turonova et al., 2020). Cryo-EM single-particle analyses (SPA) have provided molecular insights into the virus assembly and replication process (Sirohi et al., 2016; Sevvana et al., 2018), while *in situ* cryo-ET has been employed to analyze the mechanisms of virus-host attachment and invasion (Romero-Brey and Bartenschlager, 2015; Schur et al., 2016; Luque and Caston, 2020). These advances have greatly contributed to our understanding of viral infections and have direct implications for the development of vaccinations and therapeutic options. In this review, we focus on the progress in understanding the severe acute respiratory syndrome coronavirus-2 (SARS-Cov-2), the virus responsible for the devastating global COVID-19 pandemic in 2020, and the interactions with their host cells through the application of cryo-EM based technologies.

## Research of coronaviruses prior to COVID-19 era

In 2002, prior to the appearance of SARS-CoV-2, a closely related pathogenic virus known as severe acute respiratory syndrome coronavirus (SARS-CoV) emerged through zoonotic jump, resulting in widespread infections worldwide (Cherry and Krogstad, 2004). Subsequently, two more SARS-CoV-like viruses emerged from zoonotic origins, the Middle East respiratory syndrome coronavirus (MERS-CoV) in 2012 and eventually SARS-CoV-2 in 2019.

**FIGURE 1:**
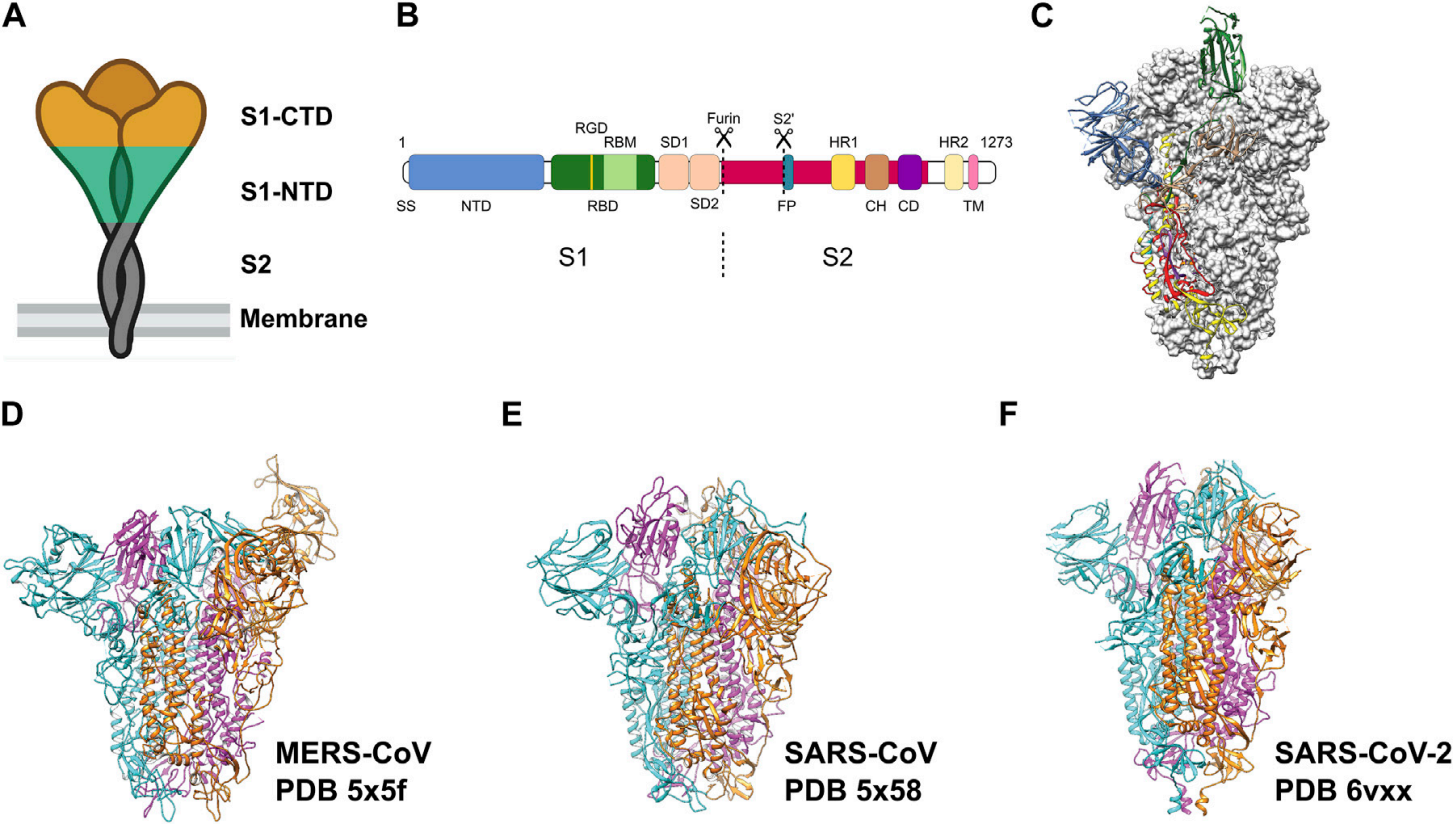
Pre-fusion structure of the S protein. **(A)** Schematic of the pre-fusion state of the trimeric spike. **(B)** Domain architecture of the S protein: N-terminal domain (NTD), receptor binding domain (RBD) with RGD motif and receptor binding motif (RBM), spike subdomain 1 (SD1), spike subdomain 2 (SD2), fusion peptide (FP), heptad repeats (HR1, HR2), central helix region (CH), connector domain (CD), transmembrane helix (TM), cytoplasmic tail (CT). **(C)** Pre-fusion structure of the ectodomain of the SARS-CoV-2 S protein. Adapted from Wrapp et al. (2020). Domains are colored as in **(B)**. Adapted from Wrapp et al. (2020). **(D–F)** Ectodomain timers of S proteins from related viruses MERS-CoV, SARS-CoV and SARS-CoV-2, respectively. Individual monomers are colored in cyan, magenta and orange, respectively.

Throughout this period, extensive research has been conducted on coronaviruses, leading to notable achievements. These include deciphering the complete genome of coronaviruses and the development of recombinant engineering techniques for studying viral structures. The application of electron microscopy, and tomographic imaging has played a crucial role in elucidating the structural characteristics of viral particles, understanding the evolution of the viruses, and providing mechanistic insights into the biological principles underlying this virus family. The spike protein (S) on the surface of the viruses protrudes outwards and mediates viral entry into the target cells. The S protein broadly has three regions: Ectodomain, trans-membrane region and an intracellular tail (Figure 1). The ectodomain comprises a receptor-binding S1 subunit and a membrane-fusion S2 subunit. Cryo-EM studies revealed that the S protein forms a homo-trimeric structure very similar to a clove-shape (Figure 1A). It showed that the S1 subunits form a trimeric head sitting on the top of the S2 subunit stalk (Kirchdoerfer et al., 2016) (Figure 1A). During infection, the S1 subunit recognizes the target receptor and the S2 subunit allows the fusion of the plasma membrane and viral membrane to facilitate the release of viral genome into the cell (Figures 1A–C). Despite the progress we made with SARS-CoV and MERS-CoV family (Figures 1D–E) (Gui et al., 2017; Yuan et al., 2017), it is crucial to continue investigating SARS-CoV-2 (Figure 1F) to comprehend its distinctive pathogenic impact on humans.

## The severe acute respiratory syndrome coronavirus-2 (SARS-Cov-2)

**FIGURE 2:**
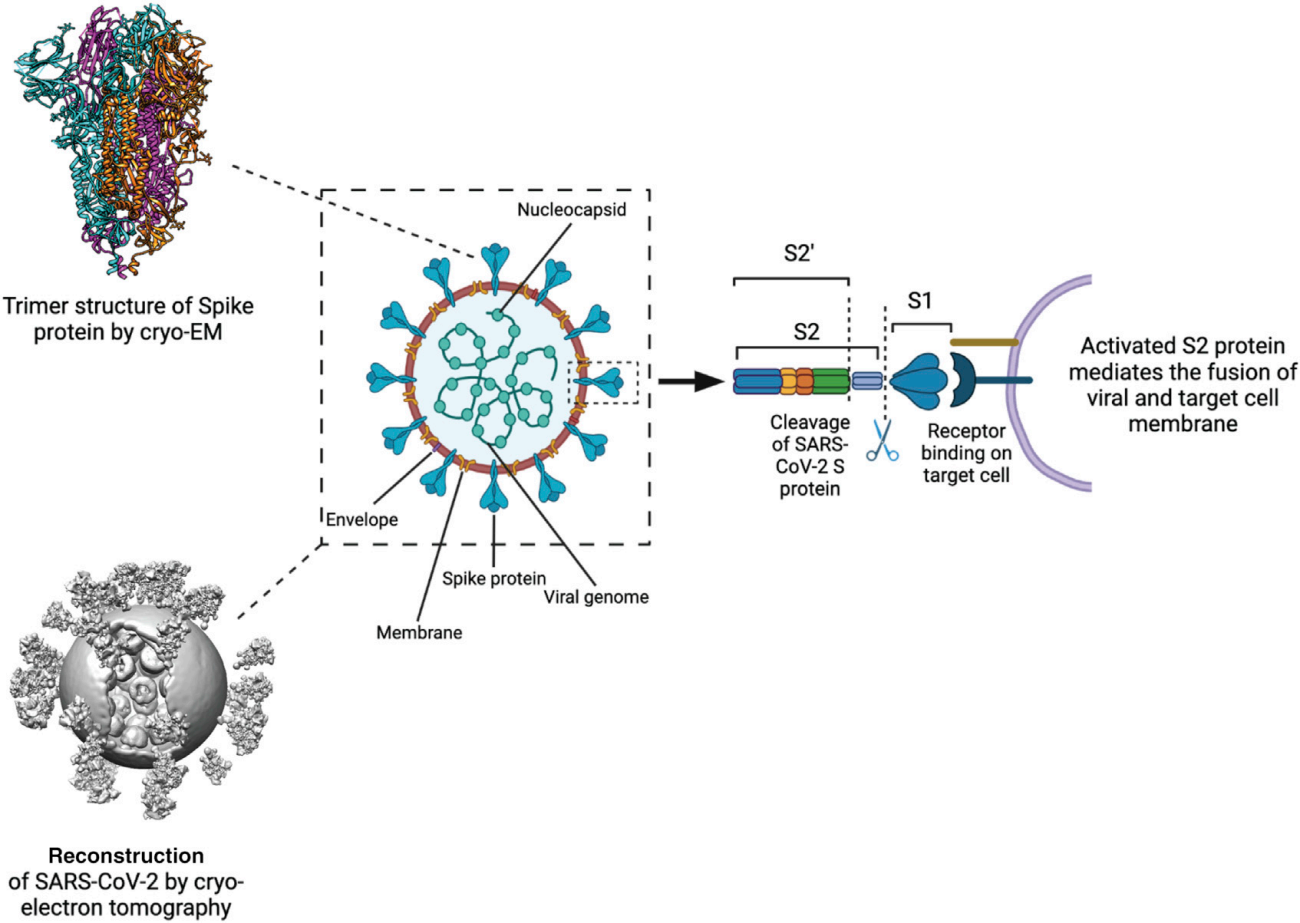
Processing and activation of SARS-CoV-2 spike before fusion. Left Structure of the S protein ectodomain (top) and a virus capsid (bottom, Yao et al., 2020). Center Schematic of the SARS-CoV2 virion. Spike in blue, membrane in brown, M protein in yellow, E protein in purple, RNP in green. Right the S protein is cut by host cell proteases at 2 positions after docking to a receptor. The process releases the S1 domain and part of the S2 domain and results in conformational change of the S2 domain and the M protein.

The coronavirus disease 2019 (COVID-19), a widely spread and devastating pandemic with huge impact on global health and economy is caused by the virus SARS-CoV-2 (Figures 1, 2). The genome of this virus was determined (Zhou et al., 2020a) and revealed that SARS-CoV-2 belongs to the *Coronaviridae* family as confirmed by the presence of 94% identical amino acid sequences used for CoV species classification. SARS-CoV-2 is classified as an enveloped, positive-sense single-stranded RNA beta-coronavirus. Similar to SARS-CoV and MERS-CoV, SARS-CoV-2 is believed to originate from zoonotic transmission (Coronaviridae Study Group of the International Committee on Taxonomy of, 2020). Genomic sequencing has revealed a 79% nucleotide homology to SARS-CoV, however, sharing a sequence homology of up to 96% with bat coronaviruses, especially the BANAL-52, BANAL-103 and BANAL-236 strains (Zhou et al., 2020b; Lu et al., 2020; Temmam et al., 2022).

Immediately after the SARS-CoV-2 genome sequence was available, cryo-EM based studies paved the way to understand the structure of the viral S protein alone or in complex with accessory proteins (Lan et al., 2020; Wrapp et al., 2020; Yan et al., 2020) (Figure 1F, 2). Similar to the other members of the SARS-CoV family, SARS-CoV-2 transfers its genome into the host cell either by endocytosis or by fusing its membrane with the host cell plasma membrane. The infection is mediated by the binding of the S protein to the angiotensin-converting enzyme 2 (ACE2) receptors on the host cell (Hoffmann et al., 2020; Xu et al., 2020). During the pandemic, several sporadic mutations in SARS-CoV-2 have emerged through recombination events which allowed the virus to spread rapidly or evade the host immune system (Rochman et al., 2021). Infections with SARS-CoV-2 can range from mild to severe, with mild cases exhibiting symptoms similar to the common cold. However, a significant proportion of individuals experience a severe course of the disease characterized by serious complications. These complications include respiratory failure, organ failure, and coagulopathic events, which can lead to life-threatening conditions and, ultimately, death (Guo et al., 2020; Huang et al., 2020).

## Architecture of SARS-CoV-2 viral particles

Studies using cryo-EM single-particle analysis (SPA) and cryo-ET techniques have provided insights into the molecular architecture of the SARS-CoV-2 viral particles (Barcena et al., 2021). SARS-CoV-2 virions have a diameter of approximately 100 nm and consist of four main structural proteins: the envelope (E) protein, the membrane (M) protein, the nucleocapsid (N) protein, and the spike (SARS-CoV-2 S) glycoprotein (Figure 2) (Ke et al., 2020; Arya et al., 2021; Laue et al., 2021).

The SARS-CoV-2 M protein is 220 amino acids long, dimerizes, and is located in the viral lipid bilayer through a triple transmembrane domain. Through its homotypic (self) and heterotypic (with other structural proteins) interactions, the M protein transforms between a compact and an elongated form to induce membrane budding of the virus (Ujike and Taguchi, 2015; Astuti and Ysrafil, 2020; Wong and Saier, 2021; Zhang et al., 2022).

The SARS-CoV-2 N protein, spanning 419 amino acids, is important for the viral assembly. It encapsulates the viral RNA and further assembles into the helical ribonucleocapsid protein (RNP) complex, which is believed to be arranged in a “beads on string” manner (Klein et al., 2020). Structurally, the N protein consists of two major domains: the N-terminal domain (N-NTD; residues 46–174) and the C-terminal domain (N-CTD; residues 247–364) separated by an intrinsically disordered region. The very ends of the M protein are also disordered (Ye et al., 2020). The N-CTD is crucial for oligomerization during genomic RNA packaging, while the N-NTD recognizes and binds to the genomic RNA (Ye et al., 2020).

The E protein of the SARS-CoV-2 is only 75 amino acids long, in which the hydrophobic transmembrane domain is positioned between two hydrophilic domains (N-terminal ectodomain and C-terminal endodomain, respectively). The E protein is responsible for the lysis and subsequent release of SARS-CoV-2 RNA during the invasion into host cells (Yang and Rao, 2021). Despite its relatively small size (8.5 kDa), the E protein forms homopentameric helical bundles and functions as a cation-selective channel (Mandala et al., 2020). These ion channels consequently disrupt the membrane potential and activate the host inflammasome (DeDiego et al., 2014).

The 1273 amino acid S protein is located on the surface of SARS-CoV-2 virions and plays a crucial role in the viral infection process. Like for SARS-CoV, the SARS-CoV-2 S protein also has two major subunits, S1 and S2. While the S1 subunit recognizes the receptor on the host cell surface, the S2 subunit mediates the fusion of the viral and host cell membranes (Satarker and Nampoothiri, 2020). The S1 subunit mainly has two well classified domains, the receptor binding domain (RBD) and the N-terminal galectin-like domain (S-NTD). Following receptor binding, the S1 and S2 subunits are cleaved by host proteases, bringing the virus closer to the target cell surface and facilitating membrane fusion. These conformational rearrangements subsequently trigger the activation of the viral membrane fusion machinery and allow the virus to release its genomic RNA into the host. This process then initiates the replication and proliferation cycle of SARS-CoV-2 (Cai et al., 2020; Walls et al., 2020) inside the host cell.

## Structural organization of SARS-CoV-2 spike protein

The SARS-CoV-2 S protein plays the most crucial role in host cell recognition and infection. Extensive research has been conducted to understand the attachment mechanism of the S protein to host cells, how it mediates cell-virus fusion, and further, how this knowledge can be exploited to development efficient vaccines and therapeutic strategies. The structural information provided by cryo-EM and cryo-ET has been vital in these investigations. Numerous structures of the SARS-CoV-2 S protein have been determined (Cai et al., 2020; Walls et al., 2020; Wrapp et al., 2020; Wang et al., 2021; Cerutti et al., 2022; Mannar et al., 2022; Makela et al., 2023), representing different regions and conformations, either in isolation or on the viral membrane surface as well in complex with its major receptor human ACE2 (Yan et al., 2020; Xu et al., 2021), providing valuable insights into the molecular function of the S protein.

While the S proteins of SARS-CoV-2 and SARS-CoV are highly conserved and share similar structures, there are some minor differences in the S protein of SARS-CoV and SARS-CoV-2, such as the presence of a unique furin cleavage site (^681^PRRAR^685^) on the S protein of SARS-CoV-2, which contributes to its increased pathogenicity or infectivity (Coutard et al., 2020). SARS-CoV-2 lacking the furin cleavage site was shown to have reduced replication or complete abrogation in mouse models of SARS-CoV-2 pathogenesis, highlighting the importance of furin site (Johnson et al., 2021).

**FIGURE 3:**
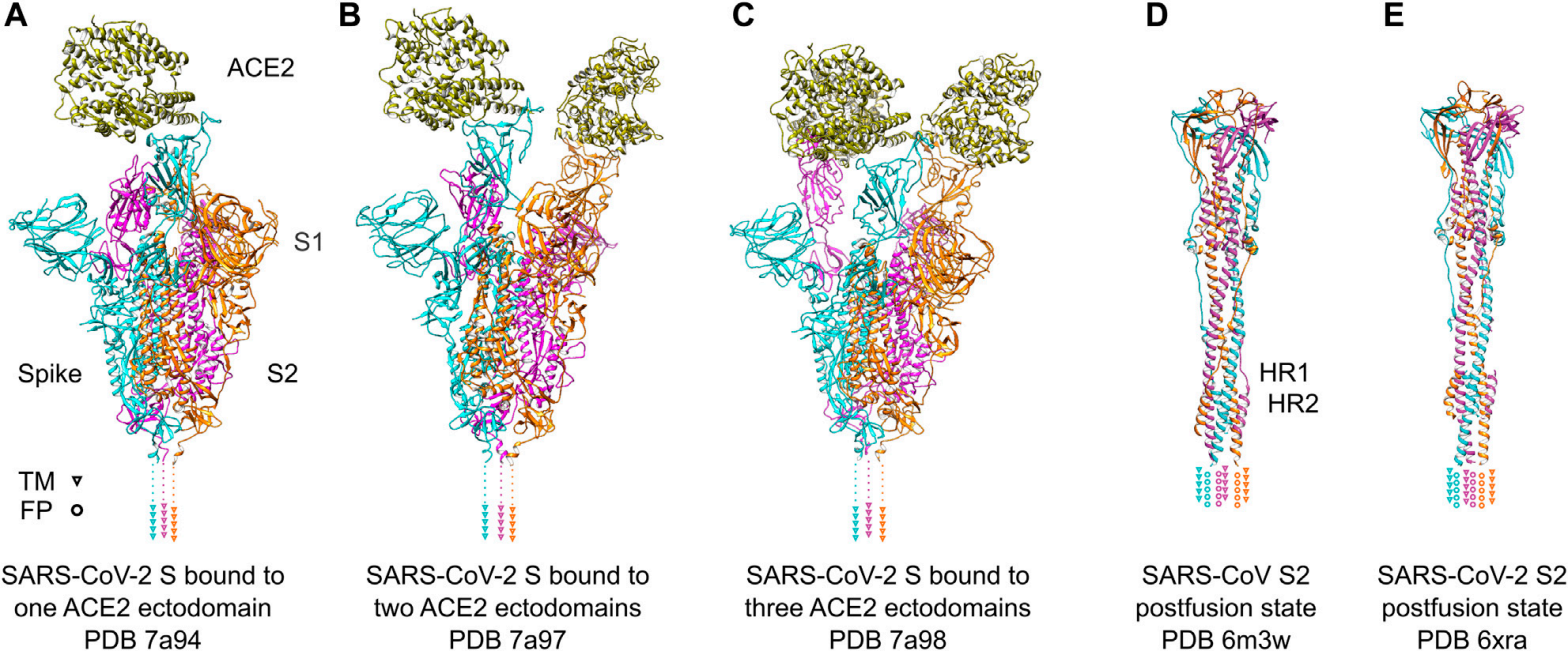
SARS-Cov-2 S protein recognizing ACE2 receptors and conformational change after fusion. **(A–C)** SARS-Cov-2 S protein trimer ectodomains bound to one, two or three ACE2 receptor ectodomains. Individual spike monomers are colored in cyan, magenta and orange, respectively. The positions of the transmembrane (TM) helices are indicated as triangles. The ACE2 ectodomain is shown in gold. **(D,E)** Postfusion state of the S2 domain of SARS-CoV and SARS-CoV-2, respectively. After a “jackknife”-motion, the TM helices and the fusion peptides (FP) are transposed to the same topological side (indicated as triangles and rings, respectively). The HR1 and HR2 domains form a characteristic six-helix-bundle. The coloring is the same as in **(A–C)**.

The S1 subunit is responsible for recognizing host cell receptors, primarily ACE2 (Figures 3A–C), while the S2 subunit facilitates the subsequent invasion by inducing conformational changes in the S protein structure (Figures 3D, E) (Zhu et al., 2021). The binding affinity of RBD and ACE2 was shown to be critical in determining the infectivity of SARS-CoV-2 (Wan et al., 2020). To promote efficient host cell entry, the S1 and S2 subunits are cleaved by the human transmembrane serine protease 2 (TMPRSS2), which cleaves the furin cleavage motif located between S1 and S2 (Hoffmann et al., 2020). Cryo-ET studies have provided valuable insights into the dynamic motion of the SARS-CoV-2 S protein on the viral surface. They have demonstrated that the S protein exhibits flexible movements facilitated by three hinges referred to as hip, knee, and ankle (Ke et al., 2020; Turonova et al., 2020; Yao et al., 2020). These hinges allow for a wide range of pivoting motions, enabling the RBD to adjust and interact efficiently with cellular receptors on the host cell surface. Notably, the S protein also displays flexibility in its attachments to host receptors (Kuhn et al., 2023). By adjusting the orientation of the RBD, the S protein can effectively engage with cellular receptors, thereby facilitating the attachment and subsequent fusion of the viral and host cell membranes. A comprehensive understanding of the dynamic behavior of the S protein is important for the development of vaccines and therapeutics targeting SARS-CoV-2. By targeting specific conformations or stabilizing the S protein in certain states, it may be possible to interfere with the virus-host interaction and prevent viral entry into host cells. Such knowledge derived from cryo-electron tomography studies can serve as a foundation for the design and implementation of strategies aimed at inhibiting viral infection and controlling the spread of COVID-19.

## SARS-CoV-2 S1 subunit—the viral grappling hook

The S1-NTD (N-terminal domain) of the S protein plays a crucial role in the initial attachment to host cells by recognizing glycans on the cellular surface (Fantini et al., 2021). However, the receptor binding motif (RBM; residues 438–506) within the RBD, which directly interacts with the ACE2 receptor, shows a lower level of conservation of approximately 50% (Wan et al., 2020). Biophysical studies showed that the RBD-hACE2 binding in SARS-CoV-2 is ten-fold higher than that of SARS-CoV (Lan et al., 2020; Wrapp et al., 2020). Structural studies have shed light on a possible mechanism how RBD-SARS-CoV-2 binding affinity is higher. The RBD domain in SARS-CoV-2 attains a more compact conformation than that of SARS-CoV, which in turn allows higher propensity for interactions with hACE2 (Shang et al., 2020). Further, specific mutations in the binding region for ACE2 evolved in SARS-CoV-2 compared to the SARS-Cov spike. E484 and F486 enable an ionic interaction with K31, and a π-π-interaction with Y83 in ACE2, respectively (Lan et al., 2020; Walls et al., 2020; Wrapp et al., 2020; Jangra et al., 2021). Consequently, these mutations result in a higher affinity of the SARS-CoV-2 RBD for the ACE2 receptor.

It is interesting to note that pseudovirus entry assays have demonstrated a lower binding affinity of the full-length SARS-CoV-2 S protein compared to the SARS-CoV S protein (Shang et al., 2020). This disparity can be explained by early cryo-EM studies, which provided insights into the conformational states of the S protein trimer. Cryo-EM maps revealed two distinct states: an open state and a closed state (Figures 3A–C). In the closed conformation, all receptor-binding domains (RBDs) are buried and inaccessible to potential binding partners. Conversely, in the open state, the RBDs face upward, exposing the receptor-binding motif (RBM) and facilitating the interaction with ACE2 on the cellular surface (Walls et al., 2020; Wrapp et al., 2020). SARS-CoV S protein predominantly exposes its RBDs in the open conformation (Gui et al., 2017), whereas the SARS-CoV-2 S protein primarily adopts the closed conformation. The reduced exposure of the RBM in the SARS-CoV-2 S protein may account for the lower binding affinity observed in the viral entry assays (Shang et al., 2020).

## SARS-CoV-2 S2 subunit—the fusion machinery

The fusion of SARS-CoV-2 virions with the host cell membrane is facilitated by the S2 subunit of the S protein. Following receptor attachment, the fusion peptide (FP) domain initiates the conformational rearrangement of the S protein, transitioning from a pre-fusion to a post-fusion state (Tang et al., 2020). The mechanism is similar for SARS-CoV and SARS-CoV-2. The postfusion arrangement of both proteins shows an rmsd of 1.234 Å for 991 residues (secondary-structure matching, trimer) and a sequence identity of 87.9% (Figures 3D, E). The fusion peptide is located within the FP domain and plays a crucial role in membrane fusion with its mainly hydrophobic amino acids. Cleavage of the S protein in SARS-CoV-2 exposes the FP domain, which contains abundant hydrophobic residues as well as potential lipid binding residues that contribute to the membrane-piercing activity of the fusion peptide, facilitating its insertion into the host cell membrane (Millet and Whittaker, 2018). The evolution of the S protein to consist of an additional furin cleavage site which are targeted by tissue wide expressed furin-like proteases allowed SARS-CoV-2 to infect organs beyond respiratory system (Hoffmann et al., 2020).

The engagement of the FP domain is aided by two heptapeptide repeat (HR) domains, HR1 and HR2, also known as the “fusion core regions” (Figures 3D, E). Both HR domains contain heptapeptide repeats (HPPHCPC) with a specific sequence motif, where “H” denotes a hydrophobic residue, “P” denotes a polar residue, and “C” denotes a charged residue (Chambers et al., 1990). These HR domains play a role in the fusion process by promoting interactions between adjacent S protein trimers. During membrane fusion, the interactions between the HR1 and HR2 domains within the S protein trimer result in the formation of a six-helix bundle structure. This structural module brings the viral and host cell membranes into close proximity, facilitating membrane fusion and the subsequent injection of the viral genome into the host cell (Du et al., 2009; Xia et al., 2020). A synthetic HR2 derived peptide can interact with the HR1 to form a stable six-helix bundle (Liu et al., 2004) and inhibit SARS-CoV and SARS-CV-2 infection. This highlights an attractive target for therapeutics and vaccines. Finally, the transmembrane domain of the S protein anchors the protein into the membrane of the virus and has been shown to be important for spike trimerization and membrane fusion (Zhu et al., 2021).

## Binding of alternative cellular receptors

The SARS-CoV-2 S protein primarily interacts with ACE2 on human cells for viral entry (Yan et al., 2020; Zhang et al., 2020). However, additional host receptors have been identified to interact with the S protein potentially enabling alternative cell entry routes, which offers an explanation as to why SARS-CoV-2 can infect different cell types ranging from lung epithelial cells, platelets, and brain glial cells.

### The S-protein and NRP-1

Following processing of the furin cleavage site, the S1 subunit of the S protein exposes a C-terminal amino acid sequence (^682^RRAR^685^), known as the “C-end rule” (CendR) motif, which plays a role in virus-host interactions (Teesalu et al., 2009). Neuropilin-1 (NRP-1), a transmembrane receptor expressed on various cell types, including epithelial cells in blood vessels, has been shown to bind the CendR motif (Plein et al., 2014). Cells expressing NRP-1 have been found to exhibit increased levels of SARS-CoV-2, despite the low expression of ACE2 (Cantuti-Castelvetri et al., 2020). The interaction between NRP-1 and the S1 CendR motif has been observed by X-ray crystallography and supported by biochemical studies (Daly et al., 2020). Mutations in the furin cleavage site of the S1 subunit or depletion of NRP-1 expression in host cells significantly reduce viral entry (Cantuti-Castelvetri et al., 2020; Daly et al., 2020). NRP1 was further shown to increase TMPRSS2-mediated entry of SARS-CoV2 (Cantuti-Castelvetri et al., 2020). In the Omicron variant, which has mutations close the CendR motif in the S protein is predicted to have more enhanced binding to NRP1 (Baindara et al., 2022).

### The S-protein and CD147

The transmembrane glycoprotein basigin (BSG) or cluster of differentiation 147 (CD147), known to mediate bacterial and viral infections, has been implicated in the viral entry of SARS-CoV. CD147 can interact with the RBD of the S protein, suggesting also a potential involvement in SARS-CoV-2 infection (Chen et al., 2005). In cellular experiments, CD147 has been shown to facilitate SARS-CoV-2 cell invasion, and its interaction with the S protein has been observed in negative staining transmission electron microscopy (TEM) experiments (Wang et al., 2020). An antibody against CD147 suppressed the replication and further infection of SARS-CoV-2 in cell lines (Geng et al., 2021). However, the CD147 and SARS-CoV-2 S protein interaction was not detected in *in vitro* binding assays (Ragotte et al., 2021; Shilts et al., 2021). These results may point towards the involvement of additional proteins for the interactions of S protein and CD147 *in vivo*. Nevertheless, the precise interplay between SARS-CoV-2 S protein and CD147 is not fully understood, emphasizing the importance of studying alternative viral entry routes (Behl et al., 2022).

### The S-protein and TLR4

Toll-like receptor 4 (TLR4), a pathogen-sensitive receptor of the innate immune system, has been associated with the recognition of the SARS-CoV-2 S protein (de Kleijn and Pasterkamp, 2003; Aboudounya and Heads, 2021). Binding of the S protein to TLR4 initiates an immune response similar to that induced by bacterial lipopolysaccharides (Shiratoand Kizaki, 2021; Zhao et al., 2021). However, further research is required to fully understand the immune cascade triggered by the interaction between TLR4 and the S protein and if it contributes to the observed cytokine storm occuring in some COVID-19 patients.

### The S-protein and RGD-recognizing integrins

Interestingly, unlike other pathogenic betacoronaviruses, the SARS-CoV-2 S protein contains an Arginine-Glycine-Aspartic acid (RGD) motif in its RBD. This motif can be recognized by several members of the integrin family, raising questions about the potential involvement of integrins in SARS-CoV-2 infection (Sigrist et al., 2020). Initial studies have indicated the involvement of integrins in the recognition of the SARS-CoV-2 S protein (Beddingfield et al., 2021; Park et al., 2021). Biochemical analyses have shown α5β1 and αVβ3 and αVβ6 integrins as potential targets for SARS-CoV-2 binding (Nader et al., 2021; Liu et al., 2022; Kuhn et al., 2023; Norris et al., 2023). Furthermore, the addition of Mn^2+^, which promotes the transition of integrins from a bent to an extended state, increases the binding of SARS-CoV-2 to cells, while RGD-mimicking inhibitors such as Cilengitide and ATN-161 reduce the interaction with cells (Nader et al., 2021; Simons et al., 2021). A recent cryo-ET study has provided the direct observation of platelet activation by the SARS-Cov2 spike, likely by the binding of the S protein to platelet integrins. The result sheds light on the underlying mechanism for rare coagulopathic events in COVID-19 (Kuhn et al., 2023).

## SARS-CoV-2 nonstructural protein (NSPs)

The SARS-CoV-2 genome encodes 16 nonstructural proteins, referred to as NSPs. These proteins play a critical role in the replication of the virus and translation of the virus proteins. Cryo-EM facilitated structural analyses of NSPs. Some notable examples include the structures of the actively replicating RNA-dependent RNA polymerase (RdRp), which consists of nsp7, nsp8, and nsp12 (Hillen et al., 2020), SARS-CoV-2 replication-transcription complex (RTC) with helicase (nsp7, nsp8, nsp12 and nsp13) (Cai et al., 2020), as well as SARS-CoV-2 RTC with an RNA binding protein nsp9 (Yan et al., 2021). These structural studies show the direct insights into the viral replication machineries, which is instrumental for drug development. These studies have advanced drug development efforts aimed at inhibiting viral replication, including those for Remdesivir (Yin et al., 2020), Suramin (Yin et al., 2021) and Molnupiravir (Kabinger et al., 2021).

## Life cycle of SARS-CoV-2

**FIGURE 4:**
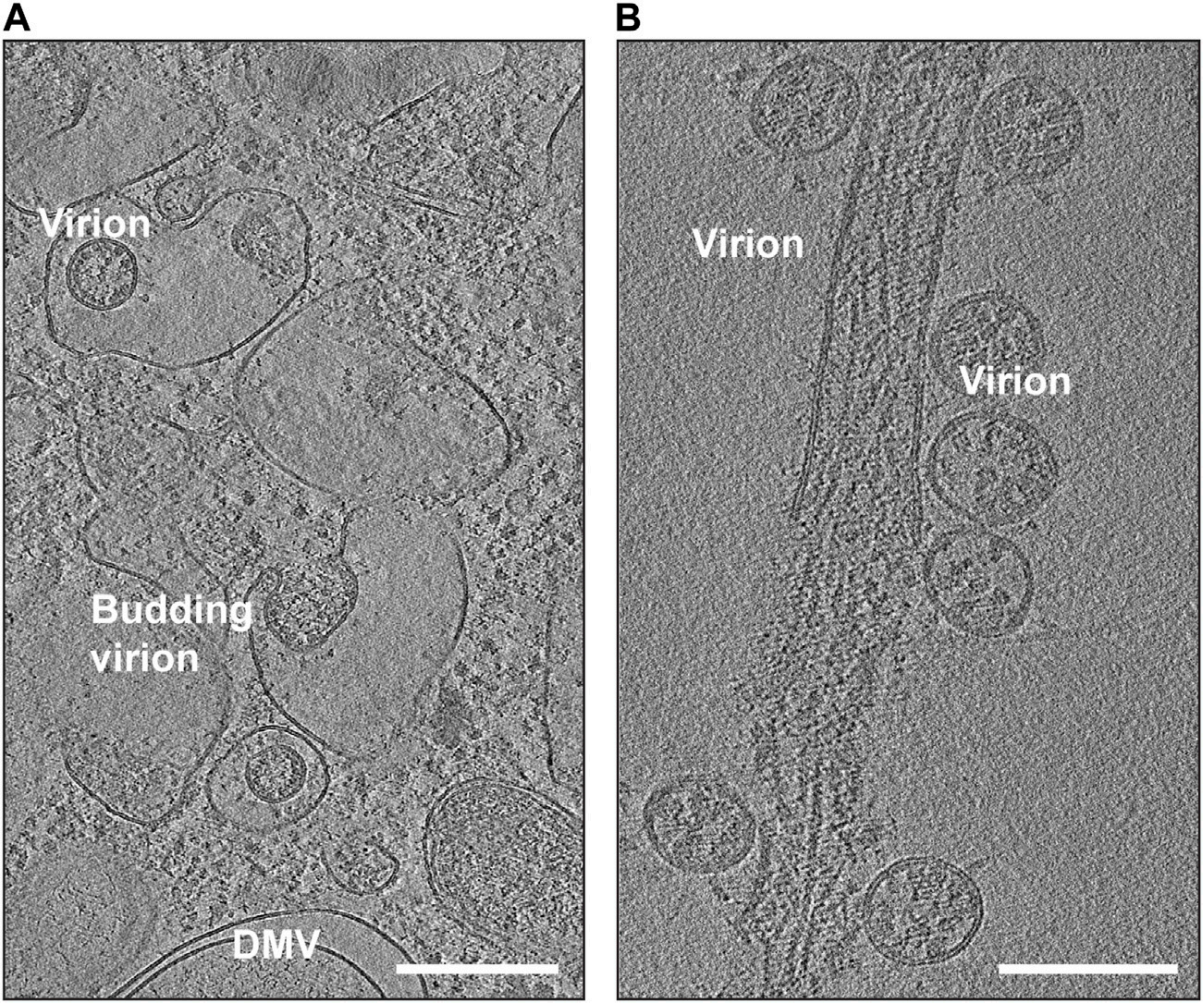
Snapshots of cryo-EM reconstructions of SARS-CoV-2 virions inside VeroE6 cells. **(A)** Budding SARS-CoV-2 virions (EMD-11863). The location of a double-membrane vesicle (DMV) is indicated. Scale Bar: 300 nm. **(B)** Release of SARS-CoV-2 virions (EMD-11867). Scale Bar: 200 nm.

SARS-CoV-2 is known to target various cell types, including those in the respiratory system, blood vessels, platelets, heart, and nervous system. Some of these cells can serve as sites for viral replication. The use of *in situ* cryo-electron tomography (cryo-ET) has been instrumental in studying the replication process of SARS-CoV-2 (Klein et al., 2020; Mendonca et al., 2021) (Figure 4). Once the virus delivers its genome to the host cell, it hijacks cellular trafficking pathways to support its replication activity (Figure 4A). Like its family members, SARS-CoV-2 uses host cell membranes to form double-membrane vesicles (DMVs) (Figure 4A), in which the viral replication process, including RNA and protein synthesis, and virion assembly takes place. Previous electron microscopic studies on SARS-CoV and MERS-CoV suggested that DMVs were derived from the cisternae of the endoplasmic reticulum (Oudshoorn et al., 2017). It is believed that the DMVs protect the viral genome from degradation by cellular ribonucleases or by detection by host immune responses (Klein et al., 2020; Snijder et al., 2020; Wolff et al., 2020). A molecular complex was identified to connect the interior of the DMVs to the cytoplasm possibly for exporting and importing of the RNA molecules (Wolff et al., 2020).

For the packaging of viral RNA, the nucleocapsid (N) proteins, located in proximity to DMVs, bind to RNAs exiting double-membrane-spanning pores and form ribonucleocapsid complexes. These complexes then oligomerize to form the viral core, encapsulating the genomic RNA (Klein et al., 2020; Wolff et al., 2020). The M proteins oligomerize on the membrane of the ER-Golgi intermediate compartment (ERGIC) and provide a scaffold for embedding the structural proteins E and S (Mariano et al., 2020; Kumar et al., 2021). Finally, the hydrophobic tail of the M protein recognizes the ribonucleic core complex, and with the support of the E proteins, completes the formation of SARS-CoV-2 particles (Luo et al., 2006; Lu et al., 2021). There are reports that the E protein directly interacts with the N protein (Tseng et al., 2014; Schoeman and Fielding, 2019), possibly also supporting the formation of virions. In support of this, expression of N protein was shown to increase the production of virus-like particles in some coronaviruses (Ruch and Machamer, 2012). However, the precise role of N and E protein interaction in viral assembly and release is yet to be determined.

Following assembly at the ERGIC, the beta coronaviruses can traffic to the Golgi apparatus, where they undergo posttranslational modifications (Fung and Liu, 2018). Similar to other enveloped viruses (Ravindran et al., 2016), it was believed that coronavirus family also uses biosynthetic secretory pathways to travel to the plasma membrane and egress by exocytosis (de Haan and Rottier, 2005; Stertz et al., 2007). However, it was recently discovered that betacoronaviruses can exploit lysosomal trafficking for their egression from host cells (Ghosh et al., 2020). This results in impaired lysosome acidification, inactivation of lysosomal degradative enzymes, and disruption of antigen presentation pathways (Ghosh et al., 2020). It is yet to be fully understood if the viruses reach lysosomes from Golgi network directly through the late endosomes (which mature into lysosomes) or through ER-phagy (lysosomes engulfing fragments of ER) or another unknown pathway. Once SARS-CoV-2 reaches the extracellular region (Figure 4B), the virions are capable of infecting other individuals through airborne transmission or by binding to host cells using their crown of S proteins.

## Conclusion

The research conducted on coronaviruses prior to the COVID-19 pandemic, along with the advances in cryo-EM technology, has played a crucial role in addressing the global public health crisis. Taking full advantage of that, the structural studies of SARS-CoV-2 using cryo-EM have provided invaluable insights and have had a significant impact on combatting the pandemic. The initial determination of the S-protein structure using cryo-EM paved the way for the development of next-generation vaccines, and subsequently, numerous other structures of different components of SARS-CoV-2 have been elucidated. The consistent performance of cryo-EM has enabled the rapid molecular characterization of the S protein, especially in response to emerging mutations. Along with the S-protein, several other important structures have inspired drug discovery attempts in both industry and academia. These structures are otherwise inaccessible by X-ray crystallography alone. Moreover, the application of cryo-ET has provided deeper insights into the mechanisms of virus-host cell interactions. These advancements in structural studies, facilitated by cryo-EM and cryo-ET, have armed the fight against the devastating COVID-19 pandemic. They have greatly contributed to our understanding of virus biology, its interactions with host cells, and the development of effective countermeasures. These scientific endeavors have had a significant impact on shortening the duration of the COVID-19 pandemic. Continuous research into fully understanding the mechanisms of proteins from viruses like SARS-CoV-2 is essential to prevent or mitigate pandemics in the future.
